# A Low Glycemic Index Decreases Inflammation by Increasing the Concentration of Uric Acid and the Activity of Glutathione Peroxidase (GPx3) in Patients with Polycystic Ovary Syndrome (PCOS)

**DOI:** 10.3390/molecules24081508

**Published:** 2019-04-17

**Authors:** Małgorzata Szczuko, Marta Zapalowska-Chwyć, Radosław Drozd

**Affiliations:** 1 Department of Biochemistry and Human Nutrition, Pomeranian Medical University, 70-204 Szczecin, Poland; 2 Clinic of Gynecology and Urogynecology, Pomeranian Medical University, 70-204 Szczecin, Poland; mzapalowska@wp.pl; 3 Department of Immunology, Microbiology and Physiological Chemistry, West Pomeranian University of Technology, 70-310 Szczecin, Poland; radoslaw.drozd@zut.edu.pl

**Keywords:** polycystic ovary syndrome, diet, antioxidant status, GPx3, uric acid, FRAP

## Abstract

Introduction: According to a review of the literature, there is a lack of data on the mechanisms that participate in the suppression of inflammation that accompanies polycystic ovary syndrome (PCOS). Additionally, the changes in oxidative status resulting from a low-calorie diet have not been studied in a group of women with PCOS, and the oxidation and reduction processes associated with PCOS have not been explained. Material and methods: The study involved 49 women who were diagnosed with PCOS according to Rotterdam’s criteria, and 24 women voluntarily agreed to a three-month dietary intervention. The dietary intervention was carried out for 3 months. Glutathione peroxidase (GPx3) activity, the Ferric reducing ability of plasma, and uric acid concentration were measured spectrophotometrically both before and after the intervention. Statistical analysis was performed with the Statistica 10.0 software package, and a Pearson’s correlation matrix was generated. Results: A lower concentration of GPx3 was observed in women with PCOS (before the dietetic intervention began) compared with the GPx3 levels in healthy women. A relationship was shown between GPx3 levels and the concentration of prolactin, insulin on fasting, and triglycerides. After the dietary intervention, increases in uric acid and GPx3 activity were noted, as well as numerous relationships between anthropometric and biochemical parameters. The ferric reducing/antioxidant power did not change significantly. Conclusions: Inhibiting the effect of prolactin (by the level of reactive oxygen species) on the activity of GPx3 could be a starting point for the increase in antioxidative stress and the development of the inflammatory state associated with PCOS pathophysiology. Following a low-calorie diet with a lower glycemic index is proposed to silence inflammation by increasing the concentration of uric acid. During GPx3 mobilization, women with PCOS have a higher demand for selenium, and its deficiencies may contribute to disordered thyroid hormone synthesis. The three-month dietary intervention did not silence redox processes in the examined group of women.

## 1. Introduction

Polycystic ovary syndrome (PCOS) is a common hormone disorder that affects women in their reproductive years, and it is being diagnosed in younger girls with increasing frequency. This is a serious problem in the societies of developed countries and has a negative influence on health, self-esteem, and the ability to have children. It is estimated that 15% of women suffer from PCOS [1]. An important factor in PCOS pathogenesis is the presence of insulin resistance, which strengthens hyperandrogenism and leads to metabolic disorders [2]. Women afflicted with PCOS are also at risk for developing type 2 diabetes, dyslipidemia, high blood pressure, and metabolic syndrome [1,2].

According to the available literature, the changes in oxidative status in a group of women with polycystic ovary syndrome (PCOS) after following a low-calorie diet have not been studied. Although an increase in oxidative stress indexes and a simultaneous decrease in the concentration of antioxidants was reported in women with PCOS [3], this group of patients had very diverse phenotypes. Oxidative stress occurs in both obese women and women with PCOS who are not obese [4,5]. Fruit, vegetables, oils and nuts present in a diet provide, apart from numerous minerals, contains antioxidants. In western diets, their amount is often insufficient to prevent free radical processes. The antioxidants and nutrients acquired from the diet to the organism through a diet include ascorbic acid, tocopherols, β-carotene, lycopene and flavonoids. They all support the removal of reactive oxygen species and prevent their transformation into more toxic forms of free radicals and peroxides. In this way, they protect the human body against many negative effects. An example of the negative influence of oxidants is the oxidative modification of low-density lipoprotein (LDL) particles, which become atherogenic as a result. A consequence of an inappropriate diet for women with PCOS is a significant disturbance of their internal defense mechanisms [6,7].

In women with PCOS, a disruption in the activity of both enzymatic and non-enzymatic endogenous antioxidants occurs; this can also be caused by hyperglycemia, which often co-occurs with PCOS and induces mechanisms that produce reactive oxygen species (ROS) [8]. In consequence, there is a disturbed balance and increased oxidative stress [9]. Changes in the lipid profile that accompany PCOS include enhance lipid peroxidation, causing the formation of malondialdehyde (MDA), hydroperoxides of fatty acids, coupled dienes, and oxycholesterols [10]. An increased fraction of LDL in the body is characterized by the tendency to form small, dense particles rich in triacylglycerols, which are easily oxidized and rendered cytotoxic to the vascular endothelium, thus increasing the risk for atherogenesis [11].

Insulin resistance often accompanies PCOS, and it leads to the increased release of insulin by pancreatic beta cells, which in turn is the cause of increased ROS and, consequently, the decreased activity of antioxidants [12,13]. It has also been reported that insulin resistance not only escalates the oxidative stress of cells but also increases the adhesion of leukocytes to the epithelium, contributing to some of the clinical complications observed in people with PCOS [14,15]. Moreover, oxidative stress contributes to the disappearance of the functionality of pancreatic beta cells, whose lack of activity intensifies insulin resistance and furthers the development of vascular complications [16].

Glucose autoxidation (glycoxidation), which results in the formation of ketoaldehydes, also increases the process of non-enzymatic glycation of proteins, causing the irreversible formation of advanced glycation end-products, the so-called AGE proteins, and toxic derivatives of oxygen ROS [17]. The oxidative activity of ROS toward proteins leads to the formation of hydroxide, alkyl peroxide, alkyl hydroperoxide, or alkoxy radicals, and these molecules consequently break polypeptide chains apart [18]. The hydroxide radical may also lead to the oxidation of non-proteinaceous compounds, e.g., carbohydrates or metal ions, which are bound to proteins and, as a result, disrupt the biological functionality of these proteins. Oxidatively modified proteins tend to form aggregates, which are resistant to degradation because they do not undergo ubiquitination and are not recognized by proteasomes [19]. The oxidative modification of lipids, proteins, and DNA leads to disturbed homeostasis and cell death via apoptosis or necrosis [20].

In diabetics, the state of metabolic balance does not fully prevent the development of complications such as macroangiopathy (premature atherosclerosis). Oxidative stress is the main factor linking hyperglycemia with the enhanced glycation of proteins, activation of protein kinase C, formation of glycosaminoglycans, and activation of the transcription factor (NF-κB) responsible for, inter alia, the development of inflammation. In this scheme, cytokines are produced, and there is an increase in the expression of adhesive molecules and an intensification of apoptosis of damaged cells. Szczuko et al. studied women with PCOS who had a high concentration of androgens, and results showed that the inflammatory state connected to TNF-α is caused by the enhanced D4 (progesterone) synthesis pathway and the omitted or limited D5 (dehydroepiandrosterone) pathway of testosterone biosynthesis [21].

The composition of intracellular antioxidative systems involve micromolecular antioxidants (vitamin C, E, coenzyme Q, carotenes, glutathione, trace elements) and macromolecular antioxidants (enzymes), such as superoxide dismutase (SOD), catalase (CAT), glutathione peroxidase (GPx3), and glutathione reductase (GRd) [22].

Enzymatic antioxidant barriers are formed by, among others, GPx3, which has an affinity for hydrogen peroxide (H_2_O_2_) and organic hydroperoxides. It is a selenium-dependent tetramer mainly present in the cytosol. In a gradual reaction, the selenium in the active center is reduced from its oxidation state of +4 to −2, and reduced glutathione (GSH) contributes to this reaction. GPx3 is the only enzyme that removes H_2_O_2_ in the mitochondria; thus, it is crucial [23].

Uric acid is the main antioxidant in human blood serum. Its elevated concentration correlates with obesity, insulin resistance, high blood pressure, heart diseases, and stroke. In humans, it is formed as a result of the degradation of purines. Because humans no longer have urate oxidase activity, uric acid is not transformed into allantoin, as occurs in other mammals. As a result of the inhibitory activity of estrogens, uric acid levels are usually lower in women, amounting to 200–400 µM. Unfortunately, elevated levels increase the risk of hyperuricemia and gout. Uric acid removes singlet oxygen, the peroxyl radical (RO_2_), the hydroxyl radical (HO), and peroxynitrite, but it does not eliminate the superoxide radical [24].

It should be noted that the antioxidative capacity of an organism is not reflected by the concentration of a single antioxidant but results from the cooperation of several compounds having antioxidative functions, plenty of which are present in plants [25,26]. The parameter determining both enzymatic and non-enzymatic antioxidants is the ferric reducing/antioxidant power (FRAP) mechanism. The aim of our study was to determine which of the antioxidants increase in activity after the implementation of a 3-month low glycemic index (GI) diet supplemented with exogenous antioxidants and essential fatty acids (EFA) to suppress inflammation [27]. The hypothesis of the study was that a low glycemic index diet rich in exogenous antioxidants can affect the suppression of inflammation caused by oxidative stress.

## 2. Results

**Figure 1:**
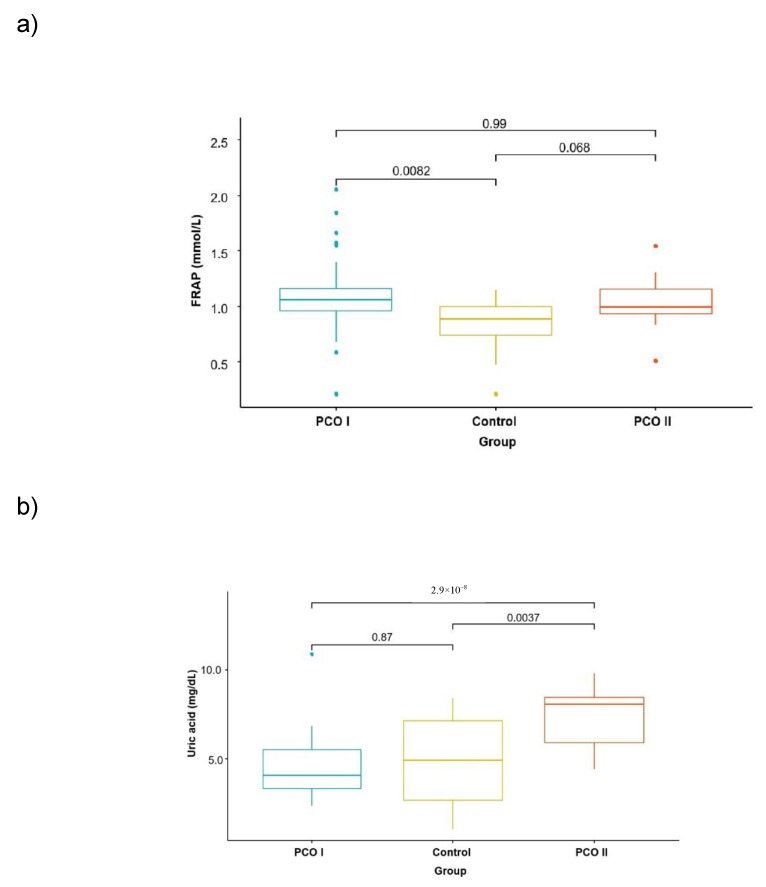

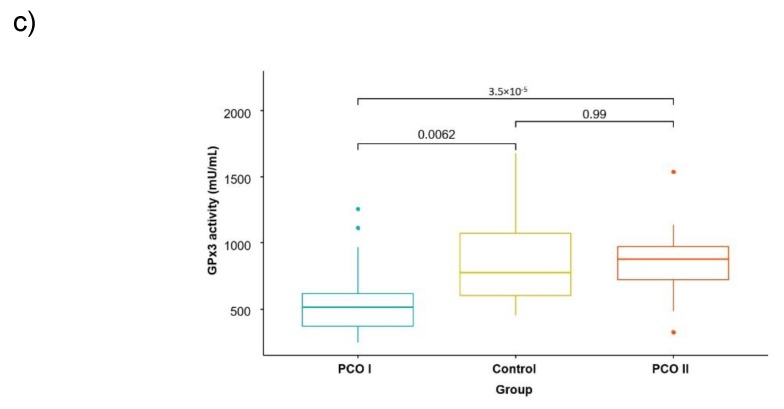
Comparison of FRAP (**a**), uric acid levels (**b**), and GPx3 (**c**) activity in the plasma of women with PCOS before dietary intervention (PCO I), women with PCOS after dietary intervention (PCO II), and women not diagnosed with PCOS (control). The means were compared using the Kruskal–Wallis test.

Statistically significantly lower GPx3 activity (*p* = 0.000) was observed in women with PCOS compared with the control group (Table 1 and Figure 1a–c). After the dietary intervention, a significantly increased level of uric acid (*p* = 0.000) and GPx3 (*p* = 0.000) was observed compared with the levels before the intervention (PCO I). The total antioxidative status did not change significantly (*p* = 0.541) (Table 1).

In women with PCOS, no significant correlations were observed between uric acid and FRAP according to the tested biochemical parameters (Table 2). There was, however, a significant correlation noted between GPx3 and prolactin, insulin measured on fasting, and triglycerides (Table 2). 

When analyzing the correlation matrix for the biochemical and anthropometric parameters of women with PCOS after the three-month dietary intervention, some significant correlations with respect to numerous parameters were observed.

Uric acid was significantly negatively correlated with such anthropometric parameters as body mass, BMI, body mass reduction, total body water, arm circumference, and thickness of the skinfolds on shoulder-blade and hip. A positive correlation was observed between uric acid and prolactin and glucose on fasting (Table 3).

For the total antioxidative status of plasma FRAP, a negative correlation was observed with relation to such anthropometric parameters as body mass, total body water and extracellular water, the content of fat tissue (kg), hip and arm circumference, and the thickness of skinfolds. A positive correlation was noted for dehydroepiandrosterone sulfate (DHEA-SO_4_) and glucose (Table 3).

The activity of GPx3 was positively correlated with body mass, BMI, the content of fat tissue (kg), hip and arm circumference, and the thickness of arm and hip skinfolds. A negative correlation was observed for muscle mass measured in kg and % and the concentration of DHEA-SO_4_ (Table 3).

## 3. Discussion

Some authors have expressed the need for using oxidative stress biomarkers in clinical diagnostics to aid in the identification and treatment of many diseases [28]. In PCOS patients, we previously observed increased oxidative stress and a reduced antioxidative capacity due to lower levels of enzymatic and non-enzymatic antioxidants, measured as the total antioxidant status (TAOS) [3] or FRAP [4]. In our own studies, we also observed decreased concentrations of uric acid and GPx3; however, we cannot deduce the reason that FRAP is found to be higher in women with PCOS in our study than in the control group. We propose that it is an effect of female hormones. Other studies have shown that the reason for reduced GSH-Px activity might be the reactions of the enzyme with lipid peroxidation end-products, such as MDA or 4-hydroxynonenal, which can lead to the inactivation of the enzyme [20].

After the dietary intervention, because GI and diet caloricity were reduced, we observed an increase in GPx3 activity and concentration of uric acid in plasma. Therefore, the reduction in post-prandial hyperglycemia, which mainly contributes to the formation of ROS, and supplementation of the diet with exogenous antioxidants led to a situation in which exogenous antioxidants played a major protective role against ROS, thus relieving endogenous antioxidative systems. Since FRAP decreased only slightly, it can be deduced that the level of free radicals was still high, despite following the diet for three months (Table 2).

**Figure 2:**
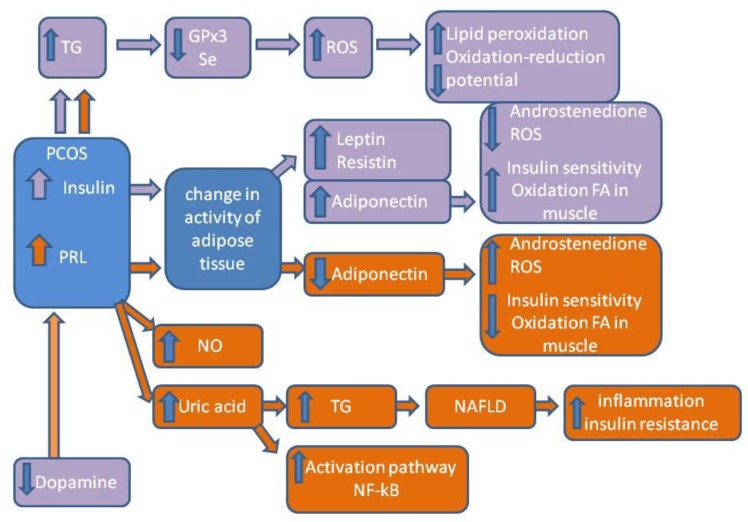
PRL and insulin impact on the development of inflammation involving ROS in PCOS.

When analyzing the correlations between biochemical parameters, we observed that the level of triglycerides and insulin on fasting had an inverse relationship with GPx3 and the level of prolactin. Higher secretion of insulin by pancreatic beta cells contributes to increased ROS [13]. Moreover, our test group was characterized by a higher amount of adipose tissue, which stores triglycerides that have gone unused in energetic processes. Triglycerides are highly susceptible to oxidative modification and have atherogenic activity [11]. Additionally, adipocytes are the site of synthesis and secretion of leptin, resistin, and adiponectin, which contribute to lipid and carbohydrate metabolism. There is proof that adiponectin is the most abundantly produced human adipokine with anti-inflammatory, antioxidative, and insulin-forming properties, and it takes part in reproduction because it reduces the production of androstenedione [29,30]. It has favorable cardiometabolic activity, such as increasing the oxidation of fatty acids in muscles, increasing the sensitivity to insulin, and reducing the levels of reactive oxygen species (ROS) [31]. Therefore, its use in treatment to regulate ovarian androgen secretion should be considered [32]. Increased amounts of visceral tissue (which mainly synthesizes leptin), also influences the endothelium of blood vessels via adiponectin (protecting the endothelium against unfavorable changes). Unfortunately, in women with PCOS, it does not fulfill such role because prolactin, which is produced in the brain and increased in PCOS, can reduce the production of adiponectin in fat tissue. The secretion of prolactin (PRL) is controlled by a hypothalamic inhibitory factor, dopamine (DA), in a short feedback loop. Prolactin released from the pituitary gland into the blood is transported through the choroid plexus to the central nervous system (CNS), where it activates dopamine neuron systems in the hypothalamus via specific receptors. Prolactin may affect the secretion of leptin, but this effect is also dependent on the length of the day. The results of several studies have reported the existence of interactions between prolactin, melatonin, and adipose tissue activity, as well as confirmed the modulating effect of the photoperiod on the formation of such interactions [33]. Other researchers also showed a positive correlation between FRAP and PRL in unexplained infertility, suggesting that increased levels of antioxidants prevent ROS-induced oxidative damage. Additionally, they observed a positive correlation between prolactin and nitrite (as a derivative of nitric oxide) in plasma, which may suggest the involvement of other protective mechanisms against lipid peroxidation and atherosclerotic changes [34]. The discussed data and literature analysis are shown in Figure 2. The lipid theory of the formation of atherosclerotic changes assumes that atherosclerotic plaques form as a result of the excessive accumulation of cholesterol in macrophages present in the subendothelial layer. The endothelium has the potential to secrete substances that have mutually exclusive effects. In proper conditions, the vascular endothelium inhibits the activation of platelets (nitric oxide, prostacyclin, C protein) and clotting processes (thromboxane, oxygen radicals, fibrinogen). It maintains the condition of blood vessel widening by not allowing for excessive permeability of vascular walls (vasoconstrictive factors: endothelin 1, angiotensin II, thromboxane, prostaglandin, oxygen; vasodilating radicals: nitric oxide, prostacyclin, hyperpolarizing agent, bradykinin). It also prevents the proliferation of myocytes, the reconstruction of vascular walls (nitric oxide, prostacyclin, transforming growth factor beta, heparan sulfate, the inhibitors of extracellular proteinases), and the adhesion and transmigration of leukocytes. When the bioavailability of nitric oxide is low, there is an excessive synthesis of substances that contract vessels and convert angiotensin I into angiotensin II on the endothelium, which results in vessel contraction, platelet activation, and the adhesion of leukocytes [35]. 

After the dietary intervention, we observed a significant correlation between the increase in the level of uric acid and, in people with lower body mass, lower BMI and lower total body water; in those with a higher body mass reduction, the increased uric acid levels were related to a greater reduction in arm circumference and the skinfolds below the shoulder-blade and above the ilium. 

Correlations between uric acid and increases in PRL and glucose on fasting were also observed. Uric acid may contribute to the accumulation of triglycerides through the enhanced synthesis of fatty acids and lipogenic pathways in the liver, leading to the development of non-alcoholic fatty liver disease (NAFLD), which contributes to the development of chronic hepatitis and resistance to insulin [36]. It seems that the level of uric acid might have changed because of the increased intake (with the diet) of purine-rich products, such as fish, legumes, spinach, and asparagus. However, the level was still within the reference levels. An increased level of uric acid, as was observed after the dietary intervention, activates the NF-κB pathway through the increased phosphorylation of IκBα: hence, uric acid is probably also a proinflammatory factor [37].

Higher FRAP was linked to a higher content of muscle tissue, DHEA-SO4, and glucose and lower total body water, extracellular water, fat mass measured by hip and arm circumference, and the thickness of the skinfold below the shoulder-blade.

It was also observed that GPx3 increased with higher body mass, BMI, and fatty tissue measured as the waist and hip circumference and the thickness of the skinfolds above the ilium and on arm. Interestingly, GPx3 increased with a simultaneous decrease in the level of muscle mass and DHEA-SO_4_. Dehydroepiandrosterone (DHEA) is an endogenous steroid synthesized in the adrenal cortex, gonads, brain, and gastrointestinal tract. The pro-oxidant effect is mediated by the induction of peroxisome proliferator-activated receptors (PPARs) when DHEA reaches pharmacological concentrations in the tissue [36]. In a previous study, treatment with DHEA significantly increased the total concentration of glutathione (17%) and GSH (22%), as well as the activity of G6PDH (51%) and GPx3 (22%) and the concentration of hydrogen peroxide (33%) [38]. Therefore, dehydroepiandrosterone improved the hepatic antioxidant reserve. Increased body weight, especially fatty tissue, is linked to an elevated inflammatory state in an organism [39,40]. Mobilization of fatty tissue after the dietary intervention resulted in slightly decreased FRAP but, at the same time, it increased the activity of GPx3, which suggests that the levels of endogenous antioxidants were stabilized. Thus, it seems probable that in women with PCOS, there is a higher demand for selenium, which is used during the mobilization of GPx3 and whose excessive use in redox reactions may result in dysfunction of thyroid hormones and their disrupted synthesis/conversion; these changes often accompany PCOS in women [41]. 

## 4. Material and Methods

### 4.1. Participants

Prior to the study, the consent of the Bioethical Commission at Pomeranian Medical University was obtained. Forty-nine Caucasian women with PCOS initially expressed an interest in taking part in the study, 24 of whom voluntarily agreed to the three-month dietary intervention. The studies excluded women diagnosed with hyperprolactinemia, congenital adrenal hyperplasia, Cushing’s syndrome, androgen-releasing tumor, and acromegaly. Only women who lost a minimum of 3 kg were taken into account.

Screening tests for PCOS were performed in The Clinic of Gynecology and Urogynecology of the Pomeranian Medical University in Szczecin, Poland. 

The assessment for PCOS was based on the 2003 Rotterdam Criteria, which require that 2 of the 3 following characteristics are experienced: irregular or no menstrual periods (breaks longer than 35 days), biochemical signs of hyperandrogenism, and/or ovarian cysts (polycystic ovarian morphology detected by transvaginal ultrasonography, presence of 12 follicles or more in one or both ovaries, and/or increased ovarian volume of >10 mL) [42]. The images were obtained using an Ultrasound Voluson 730 (GE, Pfaffing, Austria). All comparisons of the results were between 2 groups as follows:

- Women with PCOS vs. the control group,
- Women with PCOS vs. women with PCOS after dietary intervention,
- Women with PCOS after dietary intervention vs control group.

The results of biochemical analyses for the test group are presented in Table 4.

### 4.2. Anthropometric Measurements

Height, weight, waist circumference, and the thickness of skinfolds were assessed according to Railly et al. [43]. Women were fasting, dressed in light clothing, and measured with an accuracy of up to 0.01 kg and 0.5 cm. The thickness of skin-fat folds was measured using a Harpenden fold measuring tool with an accuracy of up to 0.1 mm. Total body composition was also measured under standardized conditions by tetrapolar bioelectrical impedance analysis using the BIA–101 (Akern, Florence, Italy). The method specified the density of total body water (TBW), extracellular water (ECW), intracellular water (ICW), fat mass (FM), and Muscle Mass (MM). All measurements were performed 3 times: before implementing the diet, after one month of following the diet, and after 3 months of the dietary intervention.

### 4.3. Blood Sample Collection

Blood samples were obtained from the participants under fasting conditions and collected in tubes with ethylenediaminetetraacetic acid (EDTA). Next, the blood was centrifuged within 15 min of collection at 2000× *g* for 10 min. Plasma was transferred to 1.5 mL Eppendorf tubes and frozen at −80 °C until further analyses.

### 4.4. Biochemical Measurements

Selected hormones, namely, sex hormone binding globulin (SHBG), total testosterone (T), and androstenedione (AN), were analyzed to characterize the PCOS status at the baseline. Testosterone, insulin, and SHBG were assessed by ECLIA (electro-chemiluminescence immunoassay), and androstenedione was tested by ELISA (Kobas Rosch E411). Glucose was analyzed using an enzymatic method with hexokinase. 

### 4.5. The Ferric Reducing Ability of Plasma (FRAP)

The ferric reducing capability of plasma was determined using the FRAP method according to the Benzie and Strain protocol, modified to the microplate reader scale. The assay was performed by transferring 300 μL of working reagent to a microplate cell. Then, for 5 min, the temperature was equilibrated to 37 °C. In the next step, 10 μL of the sample was added to the working temperature reagent, and after 5 min, the absorbance at 593 nm was read using a TECAN m200pro microplate reader (TECAN, Genius, Männedorf, Switzerland). The assay was calibrated using aqueous solutions of Fe^2+^ in the range of 100–1000 μmol L^−1^ (FeSO_4_·7H_2_O). The standard curve was prepared using a water solution of vitamin E (Trolox). The ratio of specific antioxidants to the total antioxidant power of an organism is the following: 60% uric acid, 15% ascorbic acid, 10% proteins, and 5% α-tocopherol and bilirubin each [44].

### 4.6. Uric Acid Assay in Blood Plasma

The uric acid content in plasma was assayed by the enzymatic method according to Tietz using a diagnostic kit (Biolabo, Maizy, France). The plasma content of uric acid is expressed in mg/dL [45].

### 4.7. Glutathione Peroxidase Assay in Blood Plasma

Glutathione peroxidase (GPx3) activity was assayed according to Pagila and Valentine using a RANSEL kit (Randox Laboratories Ltd, Crumlin, UK), adapted to the microplate reader scale. For determining its activity on a substrate, cumene hydroperoxide was used. One unit of enzyme activity was defined as the amount of GPx3 that oxidizes 1 nanomole of NADPH per minute at 37 °C and pH 7.2. The activity of GPx3 is expressed as the number of units per mL of blood plasma [46].

### 4.8. Dietary Intervention

Each woman received a 7-day menu that was adjusted to individual caloric requirements and received recommendations regarding changes to their lifestyle. The compositions of the diets were calculated using the nutrition software Dieta 4.0 (IŻŻ, Warsaw, Poland), as recommended by the National Food and Nutrition Institute [47]. In each case, the diet caloricity was reduced by ca. 600 kcal. The diet consisted of 5 meals per day. All products in the menu were specified by weight. The food products recommended in the diets were the sources of all macronutrients. The products used as the sources of carbohydrates (5 portions per day) were oatmeal, wholegrain rye bread or graham bread, brown rice, groats (wheat, millet, and buckwheat), potatoes (sporadically), and whole-meal pasta. The carbohydrate products selected for the diets were characterized by their lower glycemic index.

The following products were recommended in the diets as the source of protein (on average, 1 portion of meat and 2 portions of dairy products per day): eggs, lean meat without skin (turkey, chicken), fish (mainly sea fish, such as sole, salmon, tuna), semi-skimmed pasteurized milk and dairy products (quark, natural yogurt, and buttermilk with 2% fat), nuts and seeds (almonds, pumpkin seeds, sunflower seeds, sesame seeds, and poppy seeds), and legumes (soy, red lentils, beans, peas).

The products that were the sources of fat (2 portions per day, on average) were raw oils (rapeseed oil, olive oil), oily fruits (such as avocado), as well as nuts, fish, meat, and dairy products. Vegetables or vegetables/fruits with low GI were in every meal to supplement the diet with vitamins and minerals. 

The subjects were advised to use braising, roasting, cooking in water, and steaming as heat treatment techniques to prepare their food. Additionally, each subject was instructed to drink approximately 2 liters of fluids during the day, with herbal infusions and iodized water recommended. The average weekly ratio of energy was up to 20% from protein (the proportion of animal and vegetable protein was close to 1:1), up to 30% from fat, and ca. 50% from carbohydrates. During two control visits, the way the diet was followed was verified on the basis of a nutritional interview as well as a diary, and possible noncompliance resulted in the subject’s removal from further analyses. 

### 4.9. Statistical Analysis

The results were statistically analyzed using the software package Statistica 12.0 (Statsoft, Tulsa, OK, USA). The arithmetical mean, standard deviation, and the significance of differences (determined by ANOVA) were calculated. Normality was determined using the Shapiro–Wilk test. To assess the differences between the studied groups, the nonparametric Tukey’s test was used. The level of significance was *p ≤* 0.05. A Pearson’s correlation matrix was generated for endogenous antioxidants (uric acid and GPx3) and the antioxidative power of plasma in relation to the selected anthropometric and biochemical parameters before and after dietary intervention.

## Figures and Tables

**Table 1:** Changes in concentrations of antioxidants (uric acid, FRAP, GPx3) in the plasma of women with PCOS before and after following a low GI, low-calorie diet.

Parameter	Average Values in Groups			Significance of Differences		
	PCO I	PCO II	CG	I vs. II	I vs. CG	II vs. CG
Uric acid mg/dL	4.58 ± 1.65	7.40 ± 1.61	4.91 ± 2.72	0.000	0.542	0.001
FRAP umol/L	1093.1 ± 327.1	1041.0 ± 221.5	818.7 ± 263.4	0.541	0.001	0.005
GPx3 mU/mL	555.3 ± 220.4	858.2 ± 326.7	871.4 ± 360.3	0.000	0.000	0.902

PCO I: women with PCOS before the low-calorie diet; PCO II: women with PCOS after the low-calorie diet; CG: control group.

**Table 2:** Correlation matrix between anthropometric and biochemical parameters and antioxidants (uric acid, FRAP, GPx3) in women with PCOS.

Correlation Matrix	Uric Acid	FRAP	GPx3
Height	0.039	0.004	−0.326
BM–no clothes	−0.245	−0.138	−0.202
BMR	−0.161	−0.211	−0.003
BMI	−0.273	−0.153	−0.098
DHEA-SO4	−0.197	0.009	−0.213
Androstenedione	0.240	−0.415	0.458
TSH	0.238	−0.014	−0.155
LH	0.290	0.036	0.033
FSH	−0.072	0.203	0.374
Oestradiol	0.090	−0.188	0.225
SHBG	0.349	0.127	0.116
Testosterone	0.432	−0.101	0.097
Prolactin	−0.417	0.021	−0.656
Insulin 0	−0.002	−0.017	−0.572
Insulin after 2 h	−0.200	−0.048	−0.210
Glucose	−0.460	−0.116	−0.059
Glucose after 2 h	−0.520	−0.360	0.163
Cholesterol	−0.265	0.008	−0.419
LDL	−0.061	−0.039	−0.163
TG	−0.444	−0.011	−0.557
HDL	0.271	0.092	0.314

Values in red indicate a statistically significant correlation *p* ≤ 0.05.

**Table 3:** Correlation matrix between anthropometric and biochemical parameters and antioxidants (uric acid, FRAP, GPx3) in women with PCOS after dietary intervention.

Parameters	Uric Acid	FRAP	GPx3
Body mass	−0.833	−0.968	0.787
Body mass reduction	−0.922	−0.587	0.308
BMR	−0.540	−0.151	0.345
CPM	−0.540	−0.151	0.345
Na/K	0.142	−0.424	−0.243
TBW %	0.154	0.172	−0.496
TBW IN %	−0.024	0.543	0.040
TBW EX %	0.024	−0.543	−0.040
TBW litre	−0.662	−0.776	0.444
TBW IN litre	−0.561	−0.189	0.368
TBW EX litre	−0.363	−0.845	0.251
Phase angle PA	−0.075	0.482	0.072
fat mass %	−0.159	−0.183	0.503
fat mass kg	−0.606	−0.694	0.792
BCM kg	−0.542	−0.151	0.346
BCM %	−0.036	0.531	0.052
Muscle mass kg	0.285	0.723	−0.756
Muscle mass %	0.566	0.888	−0.877
Waist circumference	−0.447	−0.411	0.932
Hip circumference	−0.583	−0.827	0.699
Arm circumference	−0.783	−0.809	0.626
Skinfold–shoulder-blade	−0.828	−0.712	0.388
Skinfold–hip	−0.904	−0.506	0.712
Skinfold–arm	−0.314	−0.500	0.912
BMI	−0.786	−0.385	0.765
WHR	0.251	0.613	0.075
DHEA-SO4	0.296	0.701	−0.632
Androstenedione	0.297	0.228	0.407
LH	0.194	−0.164	0.580
FSH	−0.443	0.062	0.540
Oestradiol	0.520	−0.102	−0.382
SHBG	0.128	−0.257	−0.486
Testosterone	0.334	−0.372	−0.056
Prolactin	0.782	0.203	−0.197
Insulin sample 0	0.337	−0.138	0.545
Insulin after 2 h	−0.600	−0.334	0.057
Glucose	0.966	0.783	−0.468
Glucose after 2 h	−0.146	0.466	−0.392
Cholesterol	−0.223	0.163	0.455
LDL	−0.337	0.242	0.292
TG	0.247	0.158	0.544
HDL	0.285	0.0156	−0.396

Values in red indicate a statistically significant correlation *p* ≤ 0.05.

**Table 4:** Test details of women with PCOS before dietary intervention.

Parameter	Avg	SD
**Height m**	1.67	0.06
**Body mass kg**	82.97	17.32
**BMR kcal**	1508.66	146.15
**BMI kg/m^2^**	29.68	6.48
**DHEA-SO4 µg/dL**	243.29	91.44
**Androstenedione ng/mL**	3.64	1.67
**TSH mIU/mL**	1.70	0.77
**LH mIU/mL**	7.71	3.62
**FSH mIU/mL**	5.03	0.95
**Oestradiol pg/mL**	45.19	24.68
**SHBG nmol/L**	35.05	16.39
**Testosterone ng/mL**	0.73	0.96
**Prolactin ng/mL**	17.63	7.25
**Insulin 0 mU/L**	13.98	10.88
**Insulin after 2 h mU/L**	79.80	47.97
**Glucose mg/dL**	91.82	10.87
**Glucose after 2 h mg/dL**	114.53	24.45
**Cholesterol mg/dL**	179.21	30.72
**LDL mg/dL**	111.82	31.42
**TG mg/dL**	102.53	52.41
**HDL mg/dL**	55.89	18.86

## Abbreviations

GPx3	glutathione peroxidase
FRAP	ferric reducing/antioxidant power
PCOS	polycystic ovary syndrome
TG	triglycerides
DHEA-SO_4_	dehydroepiandrosterone sulphate
PRL	prolactin
AC	arm circumference
WC	waist Circumference
HC	hip circumference
FM	fat mass
TBW	total body water
BMI	body mass index
WHR	waist-hip ratio
